# Simple Approach to Mitigate the Emission Wavelength Instability of III-Nitride μLED Arrays

**DOI:** 10.1021/acsphotonics.2c00221

**Published:** 2022-05-27

**Authors:** Guillem Martinez de Arriba, Peng Feng, Ce Xu, Chenqi Zhu, Jie Bai, Tao Wang

**Affiliations:** Department of Electronic and Electrical Engineering, The University of Sheffield, Sheffield S1 3JD, United Kingdom

**Keywords:** micro-LEDs, InGaN, QCSE, microcavity, mode wavelength, distributed Bragg reflector

## Abstract

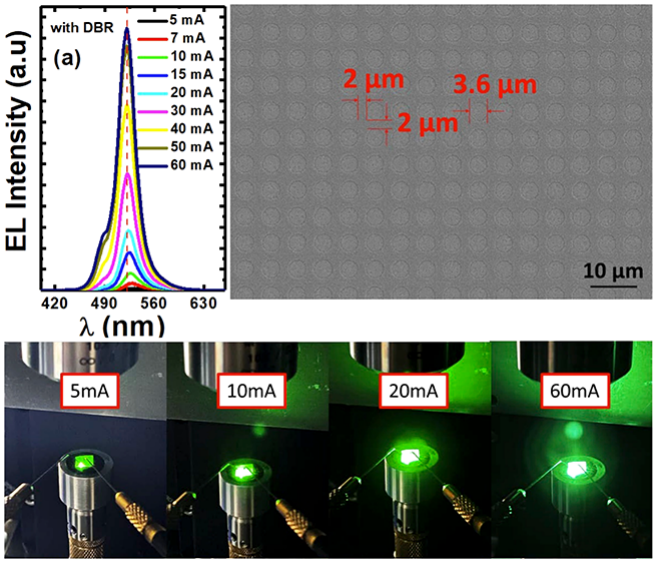

III-nitride semiconductors and their heterojunctions exhibit intrinsic polarization due to the asymmetry of their wurtzite structure, which determines all the fundamental properties of III-nitride optoelectronics. The intrinsic polarization-induced quantum-confined Stark effect leads to an emission wavelength shift with increasing injection current for III-nitride visible LEDs, forming an insurmountable barrier for the fabrication of a full color display. For instance, a yellow LED designed to produce yellow light emits green or blue light at an elevated current, while a green (blue) LED gives off blue (violet) light with increasing current. This color instability becomes a serious issue for a microdisplay such as the displays for augmented reality (AR)/virtual reality (VR) typically utilized at proximity to the eye, where human eyes are sensitive to a tiny change in light color. It is well-known that an optical mode wavelength for a microcavity is insensitive to injection current. In this work, we have demonstrated an approach to epitaxially integrating microLEDs (green microLEDs as an example, one of the key components for a full color microdisplay) and a microcavity. This allows the emission from the microLEDs to be coupled with the microcavity, leading to a negligible emission wavelength shift with increasing injection current. In contrast, identical microLEDs but without a microcavity show a large emission wavelength shift from 560 nm down to 510 nm, measured under identical conditions. This approach provides a simple solution to resolving the 30-year issue in the field of III-nitride optoelectronics.

## 1 Introduction

**Figure 1:**
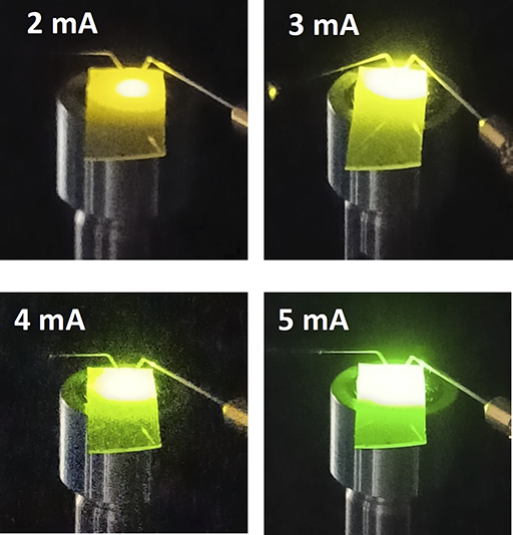
EL emission images of a III-nitride LED taken at different injection currents, demonstrating a clear color change as a function of injection current, initially yellow at 2 mA and finally green at 5 mA.

Intrinsic polarization, which determines all the fundamental properties of III-nitride semiconductors, has never been resolved since the first demonstration of InGaN-based blue LEDs with high brightness in the early 1990s.^1^ The intrinsic polarization of III-nitride semiconductors leads to piezoelectric fields induced by strain across an InGaN/GaN quantum well structure that is normally employed as an active region for III-nitride based visible emitters.^2−6^ Phenomenally, it causes an emission wavelength shift with increasing injection current along with a resultant reduction in electron–hole wave function overlap integrals causing a decreased quantum efficiency, which is the so-called quantum-confined Stark effect (QCSE).^7−10^ This becomes a very serious issue for longer wavelength LEDs such as green or yellow LEDs, since much higher indium content is required in an InGaN emitting region leading to enhanced strain that generates even stronger piezoelectric fields. It is worth highlighting that such color instability generates an insurmountable issue in the fabrication of a red–green–blue (RGB) full color display, in particular, a microdisplay such as AR/VR displays, which are typically utilized at proximity to the eye. In this case, any tiny change in color (especially human eyes are most sensitive to green) can be sensitively identified.^11^ For instance, Figure 1 shows the color change of an III-nitride LED as a function of injection current, exhibiting that it emits yellow light at 2 mA, but green light when the injection current rises to 5 mA.

Scientists have devoted considerable effort to developing semipolar III-nitride optoelectronics in the past few decades, aiming to reduce the intrinsic polarization. However, in addition to the great challenges in obtaining semipolar GaN with reasonably good crystal quality, the color instability still does not show a great improvement.^12−14^

Before we propose our idea, let us have a look at how an optical mode wavelength varies as a function of free carrier density. For a Fabry–Perot cavity, a mode wavelength that is a function of both cavity length and refractive index can be described as
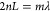
1where *L* and λ are the cavity length and the mode wavelength, respectively, *n* is the refractive index, and *m* is the order of modes within the cavity.^15^

Equation 1 means that a mode wavelength is determined by the refractive index if the cavity length is fixed.

Second, let us have a look at how a refractive index varies as a function of free carrier density. In principle, the refractive index *n* as a function of free carrier density can be expressed as^16^
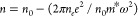
2where *n*_0_ is the refractive index when the free carrier density is zero, m* and ω are the effective mass of carriers and the frequency of emission, respectively, *n_e_* is the free carrier density, and *e* is the electron charge.

For example, if an InGaN quantum well structure is used as an emitting region for a green LED at 520 nm, eq 2 can be converted into eq 3 using m* = 0.2*m*_0_ (*m*_0_ is the free electron mass) and *n*_0_ = 2.5
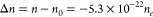
3

If a free carrier density increases from zero to a very high level, for instance, 10^19^/cm^3^, which approaches a threshold for achieving lasing,^17,18^ the change of the refractive index due to the increase in free carrier density is only about 5.3 × 10^–3^, which is very tiny. As a result, from eq 1 the shift of the resultant optical mode wavelength Δλ in the green spectral region can be estimated to less than 1 nm if the physical cavity length is below 1 μm. This is clearly negligible by comparing with the shift of the emission wavelength of current III-nitride based LEDs with increasing injection current, which is typically on a tens of nm scale, as shown in Figure 1. Furthermore, the spectral line width of III-nitride based visible emitters is generally very broad, typically >40 nm, depending on emission wavelength.^19,20^

If we can design a structure that allows the emission from a LED to be coupled into a microcavity with a physical cavity length of <1 μm, the shift of the emission wavelength can be maintained to be negligible with increasing injection current, because such an emission also obtains the feature of the optical mode via coupling. The resultant color instability can be negligible for human eyes.

Our strategy is to develop an approach to integrating a distributed Bragg reflector (DBR) and microLEDs (μLEDs) on a single chip in an epitaxial manner, where a microcavity can be naturally formed between the DBR and the air for each μLED. Very recently, we have developed a selective epitaxy overgrowth approach on a pre-patterned template, naturally forming ultrasmall and high efficiency μLEDs without involving any dry-etching process,^28,29^ which is normally used to fabricate μLEDs but induces heavy damages to the μLEDs.^21−23^

In this work, as an example for validating the above proposal, we have demonstrated that our integrated green μLEDs with a diameter of 3.6 μm exhibit a negligible shift in emission wavelength when the injection current increases from 5 to 60 mA, while identical μLEDs, but without a DBR, display a large shift in emission wavelength from 560 nm down to 510 nm, measured under identical conditions. This demonstrates that our approach provides a simple solution to resolving the 30-year issue in the field of III-nitride optoelectronics. Furthermore, our μLEDs with excellent color stability are also perfect for the fabrication of microdisplays.

## 2 Results and Discussion

**Figure 2:**
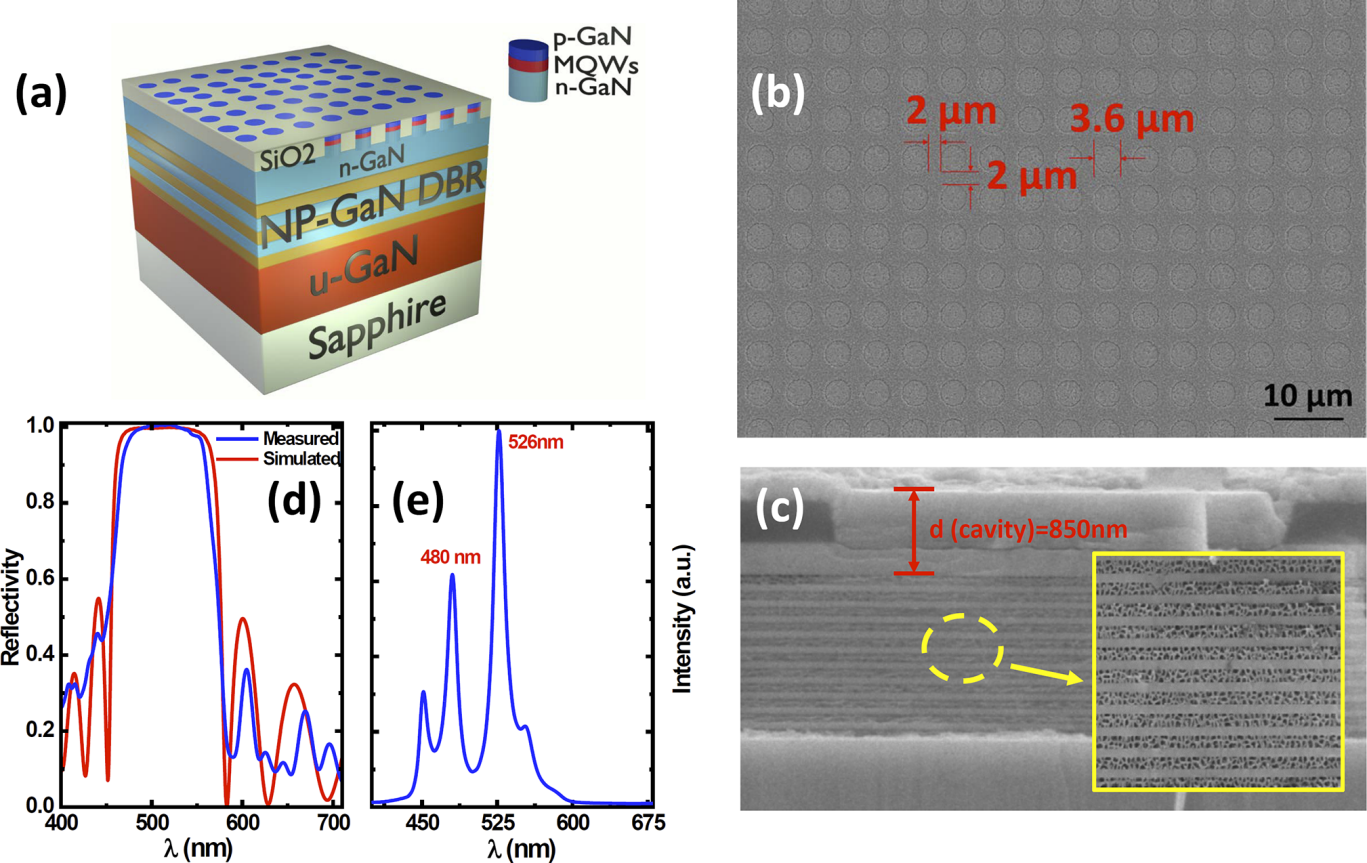
Schematic of our μLEDs with a bottom NP GaN DBR with lattice-matching (a); (b) Plane-view SEM image of our regularly arrayed μLED epi-wafer showing a diameter of 3.6 μm and an interpitch of 2 μm; (c) Cross-sectional SEM images of the μLED epi-wafer after EC etching, leading to the formation of 11 pairs of NP-GaN/undoped GaN DBR, where the inset provides a zoom-in image clearly displaying a NP GaN layer and an undoped GaN layer in each pair; (d) Reflectance spectrum of the NP GaN DBR, which agrees with the simulated results obtained by using the FDTD simulations; (e) Mode spectrum, which is obtained by using the 3D FDTD simulations to confirm the existence of optical modes.

Figure 2a schematically illustrates our design, where a nanoporous (NP) GaN based DBR with lattice-matching is used. NP GaN can be formed by means of electrochemical (EC) etching, which has been widely used to fabricate a lattice-matched DBR.^24−28^ For the details about the fabrication of the NP GaN based DBR, please refer to Methods. Such a NP GaN-based DBR exhibits a large contrast in the refractive index between the two alternating layers in each pair, that is, a NP GaN layer and an intact GaN layer.

A standard GaN layer was initially prepared on *c*-plane sapphire using a classic two-step growth approach by means of a metalorganic vapor phase epitaxy (MOVPE) technique, followed by the growth of 11 pairs of alternating heavily doped *n*++-GaN (with a doping level of 10^19^–10^20^/cm^3^) and undoped GaN, and then a 300 nm n-GaN layer with a doping level of 5 × 10^18^/cm^3^. Afterward, a 500 nm SiO_2_ dielectric film was deposited on top of the n-GaN layer by using a standard plasma-enhanced chemical vapor deposition (PECVD), followed by a photolithography process and then etching processes. The SiO_2_ layer was selectively etched down to the n-GaN surface by means of a standard inductively coupled plasma (ICP) technique. Finally, regularly arrayed microholes with a 3.6 μm diameter and a 2 μm interpitch have been formed. For the details, please refer to our recently published paper.^28,29^

Subsequently, selective epitaxy overgrowth has been carried out on the micro-patterned template by MOVPE, naturally forming regularly arrayed μLEDs, as the growth of the LED structure takes place only within the microholes due to the dielectric masks. The LED structure is standard, starting with a n-type GaN layer and then an InGaN prelayer (5% indium content), followed by 5 periods of InGaN/GaN MQWs (InGaN quantum well: 2.5 nm and GaN barrier: 13.5 nm) as an emitting region, then a 20 nm p-type Al_0.2_Ga_0.8_N acting as a blocking layer and a final 200 nm p-type GaN. The total thickness of the overgrown layers is ∼500 nm, which is level with the SiO_2_ masks. The scanning electron microscopy (SEM) image of our μLED epi-wafer, as shown in Figure 2b, exhibits that μLEDs each with a 3.6 μm diameter and a 2 μm interpitch are in a nice circular shape and an excellent uniformity.

Before device fabrication, a lattice-matched DBR was initially fabricated by conducting EC etching on part of the μLED epi-wafer, where the heavily doped *n*++-GaN layer in each pair can be converted into NP GaN but the undoped GaN remains intact. The rest of the epi-wafer is used for comparison and is denoted as μLED sample without DBR. Figure 2c displays the cross-sectional SEM image of our μLED epi-wafer after EC etching, where the inset provides a cross-sectional SEM image that is taken under high magnification, clearly showing that the DBR with a designed central wavelength at 520 nm consists of 11 pairs of NP GaN/undoped GaN layers.

In order to accurately measure the reflectance spectrum of such a NP GaN based DBR, 11 identical pairs of alternating heavily doped *n*++-GaN and undoped GaN without any further device structure on its top have been fabricated under identical EC etching processes. Figure 2d shows the reflectance spectra of the referenced DBR labeled as a blue curve, exhibiting a central wavelength at 520 nm, a high reflectivity of ∼99%, and a broad stopband of 122 nm. The measured reflectance spectrum matches a simulated result shown as a red curve in Figure 2d. A standard finite-difference time-domain (FDTD) software provided by Ansys/Lumerical is used for the simulation.

Standard device fabrication has been performed on both the μLED sample with the EC etching process (meaning with DBR) and the μLED sample without the EC etching (meaning without DBR) in the same batch. For the details, please refer to Methods. Please bear in mind that, for the μLEDs with DBR, a microcavity has been naturally formed between the DBR and the top p-type contact, which is the transparent ITO, the typical p-contact being widely used for the fabrication of III-nitride visible LEDs. Figure 2c shows that the microcavity, which was designed using a three-dimension (3D) FDTD simulation, is 850 nm long, including a 300 nm n-GaN layer, a 200 nm InGaN prelayer, 100 nm InGaN MQWs, and a final 250 nm p-type GaN layer. Figure 2e shows the simulation results, clearly demonstrating a major optical mode at 526 nm accompanied by two other modes at 480 and 450 nm, respectively, which confirms the existence of modes within the microcavity. For details, please refer to the Methods section.

**Figure 3:**
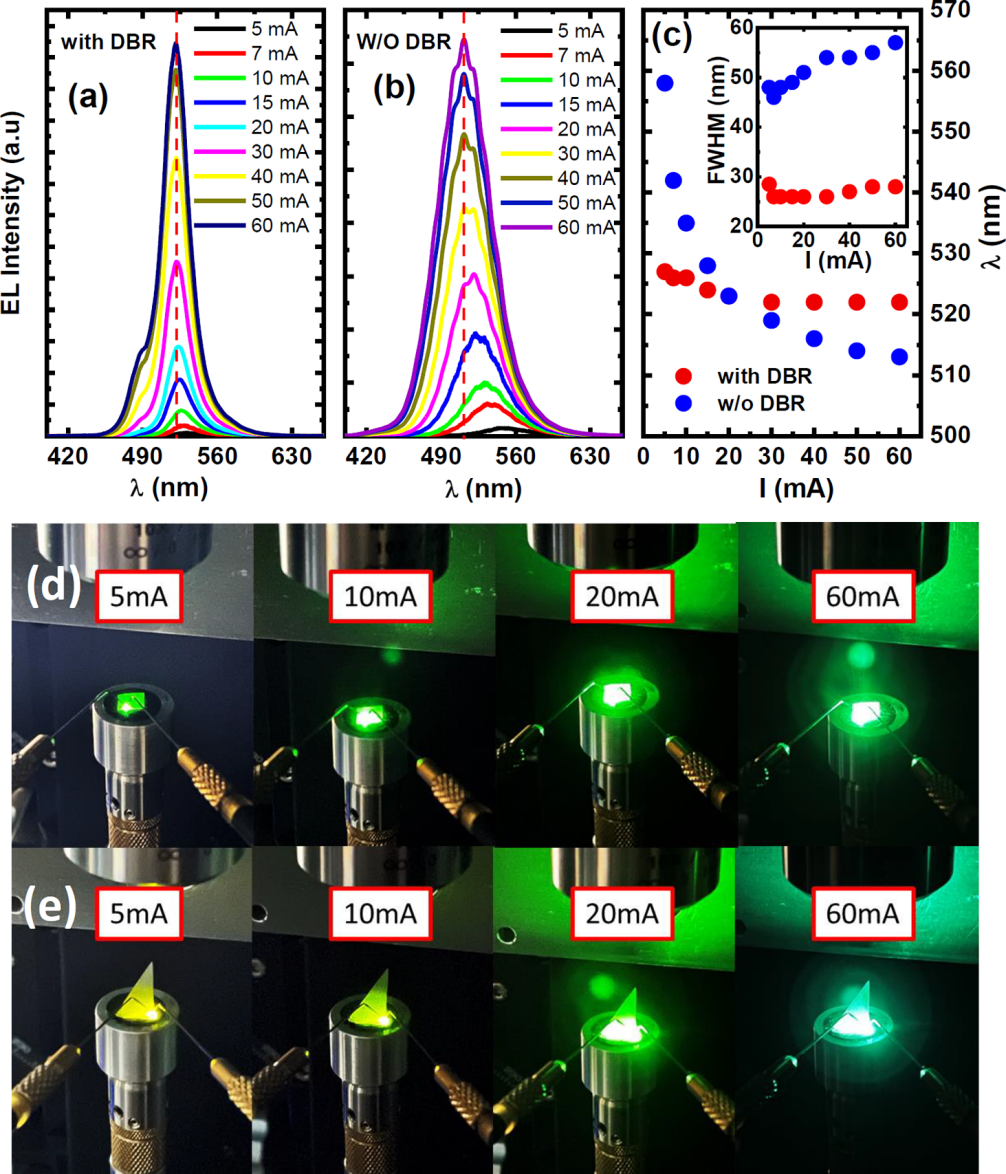
EL spectra of the μLEDs with DBR (a) and the μLEDs without DBR (b) as a function of injection current, respectively; EL emission wavelength and the full width half-maximum (fwhm) of the EL spectra of the μLEDs with and without DBRs as a function of injection current (c); and EL emission images of the μLEDs with DBR (d) and the μLEDs without DBR (e) as a function of injection current.

Figure 3a,b shows the electroluminescence (EL) spectra of the μLEDs with DBR and the μLEDs without DBR as a function of injection current, respectively. Both have been measured under identical conditions. As expected, the μLEDs without DBR demonstrated a large blue shift in emission wavelength from 560 nm down to 510 nm due to the QCSE, as mentioned above, when the injection current increases from 5 to 60 mA. In remarkable contrast, the μLEDs with DBR maintain green emission at around 525 nm under identical measurement conditions. Only at a high current injection current, a weak shoulder at 480 nm appears due to the second optical mode, which we have understood based on the simulation results, as shown in Figure 2e. Due to the mode competition, the EL spectra are overwhelmingly dominated by the emission at 525 nm. Furthermore, due to the microcavity effect, the μLEDs with DBRs show a much narrower spectral line width than those of the μLEDs without DBRs.

Figure 3d and e show the EL emission images as a function of injection current for both the μLEDs with DBR and the μLEDs without DBR, respectively, demonstrating that the light that the μLEDs without DBR emit evolves initially from yellow at 5 mA, through yellow/greenish at 10 mA, then pure green at 20 mA, and finally to green/blueish at 60 mA. Such a blue-shift in emission wavelength with increasing injection current is the fingerprint of QCSE.^30−32^ Such μLEDs cannot be used for the fabrication of a microdisplay. In contrast, the μLEDs with DBR constantly emit green light throughout all the injection current.

In detail, Figure 3c shows the EL wavelength and the full width at half-maximum (fwhm) of the EL spectra of the μLEDs with and without DBR as a function of injection current, respectively. The emission wavelength of the μLEDs with DBR remains almost at around 525 nm with increasing injection current, while the μLEDs without DBR exhibit a clear blue shift from 560 nm down to 510 nm when the injection current increases from 5 to 60 mA. The μLEDs with DBR show significantly narrower fwhm of the EL spectra than the μLEDs without DBR as a result of the microcavity effect.

## 3 Conclusion

In summary, we have proposed an integrated structure that can optically couple the emission from a LED with a microcavity, allowing the emission to obtain the properties of the optical modes formed as a result of the microcavity. This allows the LED to maintain almost constant at a designed wavelength with a negligible change with increasing injection current. By means of developing a selective overgrowth approach on a patterned template and a detailed design, we have demonstrated an epitaxial integration of μLEDs with a microcavity, leading the emission from the μLEDs to be coupled with the microcavity. Such an integrated structure displays a negligible shift in the emission wavelength of the green μLEDs with a diameter of 3.6 μm, which is maintained at 525 nm with increasing injection current, while identical μLEDs, but without a microcavity, exhibit a large shift in the emission wavelength from 560 down to 510 nm, measured under identical conditions. It is worth highlighting that such μLEDs with excellent color stability are perfect for the fabrication of a full color microdisplay.

## 4 Methods

### Nanoporous DBR Fabrication

Once the μLED epi-wafer is ready, a standard electrochemical (EC) etching technique is carried out on the μLED epi-wafer to form a NP GaN-based DBR structure in acidic solution. EC etching consists of two chemical reaction steps, namely, initial oxidation and subsequent dissolution in an acidic electrolyte under bias,^24−28^ where GaN is first oxidized in the acidic electrolyte by injected holes and is then chemically dissolved, leading to the formation of NP GaN. Therefore, EC etching can only occur to GaN with high conductivity (which can be obtained via heavily silicon-doped *n*++-GaN with a doping level of 10^19^–10^20^/cm^3^), while undoped GaN remains intact. As a consequence, the EC etching can convert the pairs of *n*++-GaN and undoped GaN into the pairs of NP GaN and undoped GaN. The EC process was performed in 0.3 M nitric acid solution under 5.5 V bias, where an indium contact as an anode and a platinum plate as a cathode are used, respectively.

### FDTD Simulation

Standard 3D FDTD simulations have been performed to confirm the existence of modes within a microcavity. The electric field is injected by a plane wave source, with an emission wavelength from 400 to 700 nm placed above the μLED structure. The geometrical data of our devices for the simulation are from the cross-sectional SEM image, which is provided in Figure 2c. As a result of considering the electric field penetration into the DBR, the effective cavity length is >1 μm for the microcavity with a physical cavity thickness of 850 nm. Time monitors placed inside the microcavity allow to determine the decay slope in the electric field, which is used to determine the wavelengths of the resonant modes. Frequency-domain power monitors are used to record the reflectance and the electric field (Ez) within the microcavity. In order to reduce the simulation memory requirements, boundary conditions are set as periodic on *X*, *Y*, and PML in the *Z* dimension.

The effective index of the NP GaN can be estimated from the volume average theory (VAT)
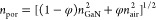
4where *n*_por_, *n*_GaN_, *n*_air_, and φ are the effective refractive index of the NP GaN, the refractive index of intact GaN, the refractive index of air, and porosity, respectively.^33^ The porosity is 0.6, determined from the SEM image, as shown in Figure 2c, giving *n*_por_ = 1.75.

### Device Fabrication

A standard device fabrication process has been employed to fabricate the μLEDs with and without DBR, both with a typical area of 330 × 330 μm^2^. Each device consists of a few thousands of 3.6 μm μLED arrays connected. Transparent indium–tin-oxide (ITO), which is prepared by means of an electron-beam deposition technique and then undergoes an annealing process in air at 600 °C for 1 min, is used as a p-type contact, while Ti/Al/Ni/Au alloys as a n-type contact are used. Both p-type and n-type electrodes are Ti/Au alloys. Device characteristics have been performed on bare chips, meaning that there is no extra process that is normally used for enhancing the extraction efficiency, such as coating, passivation, epoxy, or reflector.
